# Differential Effects of Single-Dose Escitalopram on Cognitive and Affective Interference during Stroop Task

**DOI:** 10.3389/fpsyt.2014.00021

**Published:** 2014-02-25

**Authors:** Christoffer Rahm, Benny Liberg, Maria Kristoffersen-Wiberg, Peter Aspelin, Mussie Msghina

**Affiliations:** ^1^Department of Clinical Neuroscience, Karolinska Institutet, Stockholm, Sweden; ^2^Department of Clinical Science, Intervention and Technology, Karolinska Institutet, Stockholm, Sweden

**Keywords:** cognitive control, 5-hydroxytryptamine, serotonin uptake inhibitors, magnetic resonance imaging, prefrontal cortex

## Abstract

**Background and objective:** Our aim was to study the regulatory role of serotonin [(5-hydroxytryptamine (5-HT)] on two key nodes in the cognitive control networks – the anterior cingulate cortex (ACC) and the dorsolateral prefrontal cortex (DLPFC). We hypothesized that increasing the levels of 5-HT would preferentially modulate the activity in ACC during cognitive control during interference by negative affects compared to cognitive control during interference by a superimposed cognitive task.

**Methods:** We performed a functional magnetic resonance imaging investigation on 11 healthy individuals, comparing the effects of the selective 5-HT reuptake inhibitor escitalopram on brain oxygenation level dependent signals in the ACC and the DLPFC using affective and cognitive counting Stroop paradigms (aStroop and cStroop).

**Results:** Escitalopram significantly decreased the activity in rostral ACC during aStroop compared to cStroop (*p* < 0.05). In the absence of escitalopram, both aStroop and cStroop significantly activated ACC and DLPFC (*Z* ≥ 2.3, *p* < 0.05).

**Conclusion:** We conclude that escitalopram in a region and task specific manner modified the cognitive control networks and preferentially decreased activity induced by affective interference in the ACC.

## Introduction

The cognitive control of behavior refers to a set of executive functions that enables a person to maintain goal directed activity in a changing environment by using continuously updated problem solving (1, 2). Impaired cognitive control of behavior is characteristic of a number of psychiatric disorders, such as depression (3) and schizophrenia (4).

Variations of the Stroop task have been extensively used to investigate cognitive control in humans (5, 6). The Stroop task requires that a person can maintain focused attention and is capable of continuous problem solving in the presence of conflicting or distracting stimuli. The counting Stroop task (cStroop) is a version of the task that was created for use in brain scanning experiments (7). In the cStroop, a person reports the number of words presented on a screen. The words represent a numeral and the conflict depends on whether the number of words reflects the numeral content. The cStroop was also further developed into a version with distracting affective stimuli – the affective counting Stroop task (aStroop) (8).

Using the Stroop tasks, two brain areas in particular have been identified as mediators of cognitive control: the anterior cingulate cortex (ACC) and the dorsolateral prefrontal cortex (DLPFC) (9). The ACC appears to be involved in detecting errors in form of interfering stimuli (10, 11) while the DLPFC appears to be involved in resolving cognitive conflicts through the use of focused attention and problem solving (12).

The neural activity during Stroop tasks in these two regions are found to be deranged in a number of psychiatric disease states. In a study on unipolar depression, both ACC and DLPFC were hyperactive during a version of cStroop compared to healthy controls (13). Healthy controls compared to patients with euthymic bipolar disorder exhibited relatively increased activation in middle frontal gyrus (14). Schizophrenia subjects showed significantly increased pattern of activation in the ACC (15). In obsessive compulsive disorder, the ACC showed weaker activation during a version of the cStroop, compared with healthy controls (16).

Several lines of research suggest that serotonin (5-hydroxytryptamine, 5-HT) is involved in the regulation of cognition in the prefrontal cortex (17). Research conducted in non-human primates and rodents suggests that 5-HT in the prefrontal cortex plays a modulatory role in spatial working memory (18). Also, it is critical for cognitive flexibility; when depleted it results in perseverative behaviors (19, 20). In addition, 5-HT is relevant for behavioral inhibition since elevated or reduced prefrontal 5-HT level is followed by deficits in impulse control (21, 22). 5-HT is proposed to be linked especially to those aspects of impulsivity that involves emotionally salient rewards or feedback (23).

Recent studies suggest 5-HT is one of the central neurochemical regulators of the cognitive control network (24, 25). Both key nodes in this network – the ACC and the DLPFC – are innervated by ascending raphe nuclei 5-HT projections; the ACC more densely so than the DLPFC (26). Decreasing the availability of 5-HT, using low tryptophan diets, increased the neural activity both in the ACC and DLPFC during the cStroop as measured by the blood oxygen level dependant (BOLD) response (27). Also, decreased availability of 5-HT increased the impact of affective distracting stimuli during aStroop as measured by increased reaction times (28). However, the corresponding brain imaging data for aStroop did not show any significant effects in terms of BOLD response in the ACC or DLPFC. Conversely, a 2-week administration of a selective 5-HT reuptake inhibitor agent (SSRI), increasing the availability of 5-HT, decreased the neural activity in the ACC during cStroop in a cohort of depressed patients (29).

The antidepressant effects of SSRI usually take 2 or 3 weeks of continuous treatment to become evident (30). However there is evidence of positive treatment effects on mood regulation the first 5 days (31). In fact, two studies have documented the effects on emotional and cognitive functioning after a single-dose of SSRI (32, 33). Healthy volunteers became more sensitive to fearful faces, while the opposite effect was noted among the depressed subjects. Against this background, we suggested a correlation between the level of 5-HT availability and the neural activity in the key nodes of the cognitive control network during Stroop tasks, especially for aStroop in the ACC. We hypothesized that increasing the availability of 5-HT with an acute dose of SSRI reduces affective activation in the cognitive control circuit.

We set up an experiment in which we used functional magnetic resonance in conjunction with escitalopram, a highly selective SSRI that unlike other SSRI agents has affinity only for the 5-HT transporter (34), to study the acute effects on neural activity in ACC and DLPFC among healthy controls during their performance of the cStroop and aStroop tasks and analyzed the data using both a cluster corrected whole brain analysis and a voxel corrected region of interest (ROI) analysis.

## Materials and Methods

### Participants

This case–control study was initially a pilot study where 11 healthy subjects (eight males, three females; mean age: 34.9 years, SD: 7.8 years) were recruited by local advertisement. All participants were investigated with the Edinburgh Handedness Inventory and were right handed. We used questionnaires (the Structured Clinical Interview for Diagnostic and Statistical Manual of Mental Disorders, 4th edition – SCID-1, and the Alcohol and Drug Use Disorders Identification Tests respectively – AUDIT and DUDIT) and a structured clinical interview to rule out any history of neurologic or psychiatric illness, including substance abuse. The ethics committee of the Karolinska Institutet approved the study and the participants signed informed consent after the procedures had been fully explained.

### Experimental design

Off camera, prior to the start of the experiment, the subjects were instructed how to perform the Stroop tasks and completed a computerized, 1-min training version. Each subject was then scanned on two occasions the same day, each session included both aStroop and cStroop tasks. The second scanning session was performed 4 h after ingestion of a tablet of 10 mg of escitalopram, corresponding to the expected maximum of plasma concentration of escitalopram. The subjects were blinded to the content of the tablet and were informed that it could be either a psychoactive drug or placebo.

**Figure 1:**
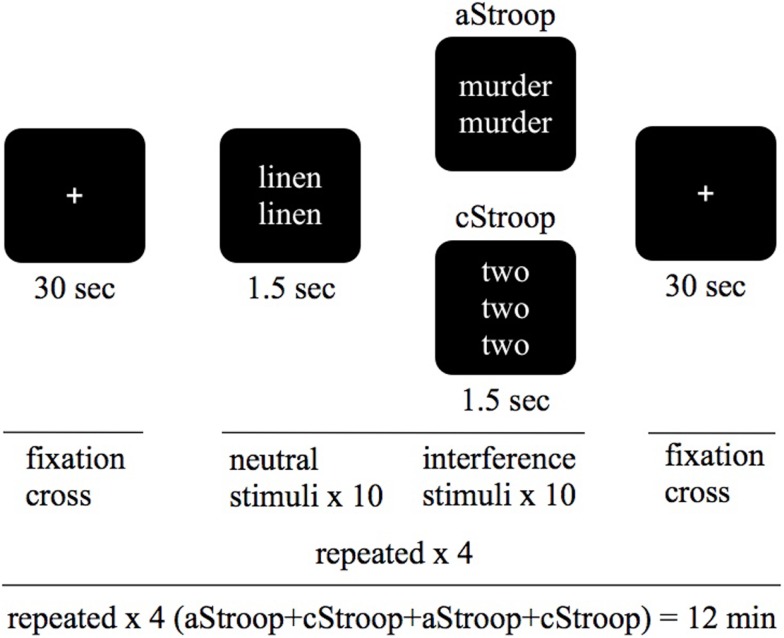
**Study design**. We used a combined Stroop paradigm: affective counting Stroop (aStroop), and cognitive counting Stroop (cStroop) intertwined. The test subjects were examined in two scanning sessions, the second one 4 h after intake of escitalopram (corresponding to C-max). The subjects were blinded to the tablet, and they were instructed that they were to see sets of one to four identical words appearing on the screen and that they should report, via button-press, the number of words in each set, regardless of what the words are.

The Stroop tasks were programed in E-Prime v 1.0 (Psychology Software Tools, 2002). A mixed block design was used. Every block consisted of a series of 10 words written in white letters against a black background. The words were presented one at a time with an interval of 1.5 s. Each word was shown in one to four copies. The subjects were instructed to consecutively report by button-press the number of copies. See Figure 1.

The cStroop (7) and aStroop (8) were programed in E-Prime v 1.0 (Psychology Software Tools, Sharpsburg, PA, USA) in a mixed block design consisting of 10 trials of 10 categorized interference words and 10 neutral words. This was repeated four times in which aStroop and cStroop blocks randomly alternated twice. Each trial began and ended with a 30-s period with a fixation spot. The words were presented in white letters every 1.5 s against a black background and stayed on the screen for 1.0 s. A black screen was shown between the two stimuli. During both aStroop and cStroop, subjects reported by button-press the number of words (one to four) appearing on the screen, regardless of word meaning. Neutral word-control trials (e.g., “table” written three times) appeared intertwined with interference trials containing number words that are incongruent with the correct response (e.g., “two” written four times). aStroop contained the same kind of neutral word-control trials, while interference trials contained emotional words (e.g., “death” written three times; Figure 1). The interference words were emotionally charged words (in the aStroop task) or numerals (in the cStroop task). The neutral and affective words were chosen from the word database affective norms for English words (ANEW) (35), which is a compilation of words characterized on the affective dimensions of valence, arousal and dominance, and on the discrete emotional categories of happiness, sadness, fear, disgust, and anger. The neutral words denoted household articles. As affective words we chose those with the highest emotional valence and arousal in the categories of sadness and fear. The words were translated into Swedish, taking into account the number of syllables and the length of the words. The same neutral and affective words never appeared twice in the same session and the presentation order was randomized. The numerals (“one,” “two,” “three,” or “four”) were chosen randomly (with help from www.random.org), but congruent combinations of numerals and number of copies were excluded. No feedback of the responses accuracy or reaction time was given to the participants.

We decided not to have a placebo arm since we assumed that the placebo effect would be similar in both aStroop and cStroop and therefore could be ruled out when comparing the two.

### Data acquisition

Participants were scanned at the Karolinska University Hospital Huddinge, in a 3-T Siemens TrioTim, with a quadrature head coil. They were instructed to avoid psychoactive substances the same morning as the day of scanning and the night before. A T1-weighted anatomical scan (MP-RAGE, 128 slices, repetition time (TR) 2400 ms, echo time (TE) 3.44 ms, with a voxel size of 1.3 mm × 1.3 mm × 1.3 mm) was obtained for each subject, which took about 6 min. We then did functional scanning while subjects performed the experimental tasks, and this took about 12 min. BOLD sensitive T2*-weighted echo planar images were acquired comprising 28 slices covering the whole brain. The voxel resolution was 1.96 mm × 1.96 mm × 4.80 mm. The TR was 1.5 s. The TE was 30 ms, and the flip angle was set to 90°. We chose to divide the total experimental session into four separate 3-min-runs (one Stroop task in each) in order to minimize habituation, and to achieve two pairs of runs for investigations of the intrasubject variability. Each run consisted of a total of 120 volumes. The first six (dummy) volumes of each run were discarded to allow for T1 equilibration effects. In total, the subjects were in the magnetic resonance imaging (MRI) scanner for 25–30 min. To rule out radiological signs of pathology, a senior consultant in neuroradiology (Maria Kristoffersen-Wiberg) evaluated the anatomical scans of each subject.

### Image analysis

All statistical analyses were done in the FSL suite. Statistical parametric mapping was performed using the FSL 4.1.5 software (FMRIB, Oxford University, UK) and data processing was carried out using FMRI expert analysis tool (FEAT) Version 5.98. Each subjects run was corrected for head motion using MCFLIRT (36). Sharp spikes of motion were removed using fsl_motion_outliers and non-brain tissue was cleaned using BET (37). To account for time difference in slice acquisition, we performed slice-timing correction using Fourier-space time-series phase shifting. To compensate for anatomical variability after registration and to permit application of Gaussian random field theory for the corrected statistical reference, the functional data was smoothed using a Gaussian kernel that was set to full-width half-maximum (FWHM) 8 mm. The grand-mean intensity of the entire four dimensional dataset was normalized by a single multiplicative factor, and physiological noise was filtered using a high-pass temporal filter set at 100 s. The parameter estimates (PE) were calculated for all brain voxels using the general linear model and the contrast images (COPE) were calculated by comparing the interference condition with the neutral condition. The time-series statistical analysis was carried out using FMRIBs improved linear model (FILM) with local autocorrelation correction (38). Registration to high resolution structural and MNI152 (2 mm) standard space images was carried out using FLIRT (39, 40).

We compared each subject’s activation before and after medication using a paired *T*-test. We compared both types of Stroop tasks in a paired *T*-test. We used a mixed effect analysis (FLAME 1 and 2) (41, 42). The *Z* statistical map was thresholded using a cluster based method for multiple comparisons correction. Cluster forming threshold was predetermined at *Z* ≥ 2.3 and each cluster was tested for a significance at *p* = 0.05 (corrected, using a Gaussian random field theory).

*Post hoc*, we also did a hypothesis driven ROI investigation in restricted sets of voxels; these voxels corresponded to the ACC and the middle frontal gyrus, which was used as a proxy for DLPFC. The ROI masks were derived from the probabilistic Harvard-Oxford Cortical Atlas. Masks were unthresholded. The *Z* statistical map was thresholded at the voxel level with the significance threshold set to *p* = 0.05 (corrected, using Gaussian random field theory).

## Results

### Whole brain analyses

**Figure 2:**
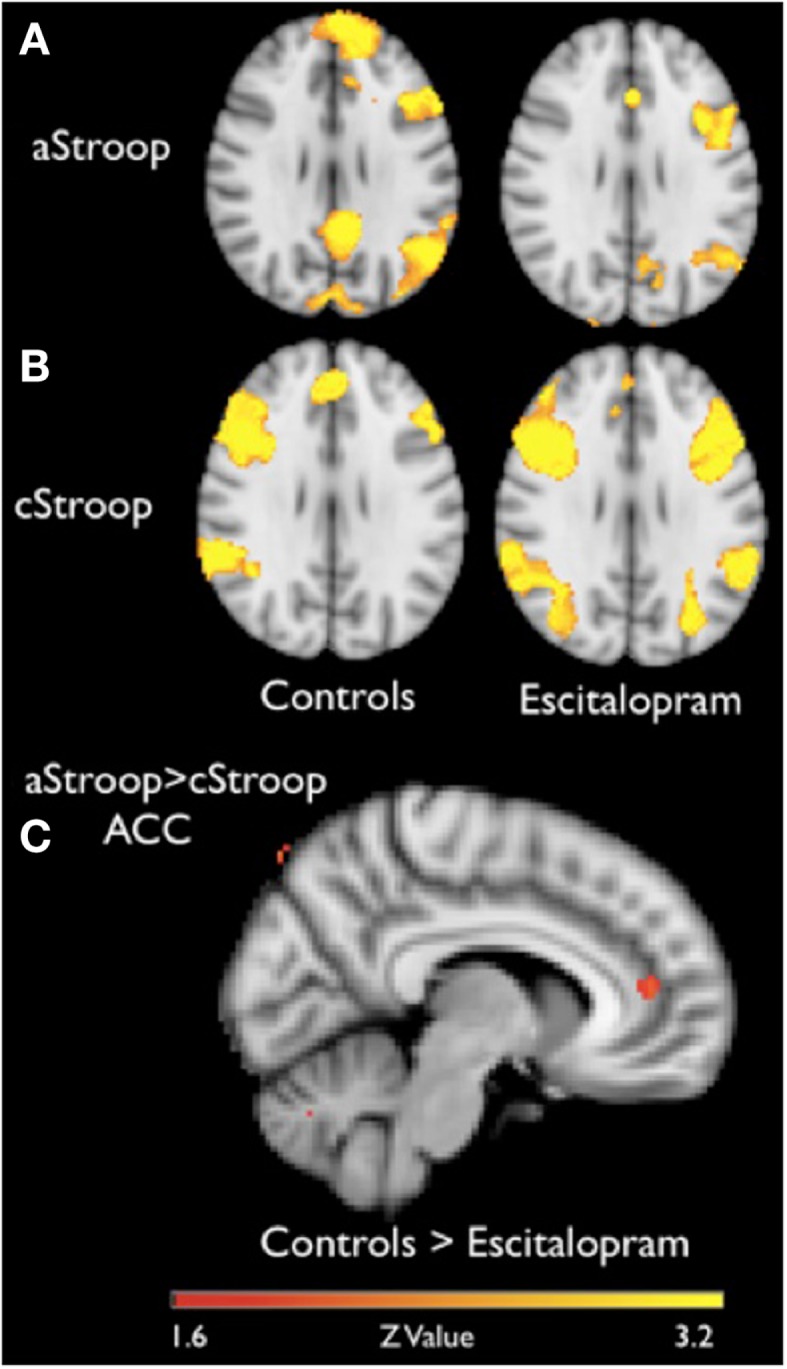
**fMRI results**. **(A,B)** Brain activation during affective counting Stroop (aStroop) and cognitive counting Stroop (cStroop), before (controls) and after intake of escitalopram (cluster forming threshold *Z* ≥ 2.3, corrected *p* = 0.05, MNI *z* = 29). In both Stroop conditions, there are significant activations in anterior cingulate cortex (ACC) and dorsolateral prefrontal cortex, key nodes in the neural cognitive control networks. **(C)** A region of interest analysis of the ACC, where aStroop activates more than cStroop before compared to after drug intake. For display purposes the cluster shown in the image has an uncorrected threshold of 0.005, but the peak voxel (*x, y, z*: 6, 44, 12) survived a corrected threshold of *p* = 0.05 (at the voxel level, using Gaussian random field theory).

In the absence of escitalopram, aStroop significantly activated two large clusters of voxels, including local maxima in left occipital cortex (*x, y, z*: −56, −70, 22), left middle temporal gyrus (−62, −54, −2), left frontal pole (−24, 54, 36), left middle frontal gyrus (−44, 24, 40), left precuneus cortex (−2, −60, 10), left cingulate cortex (−6, −50, 28), and left amygdala (−20, −12, −16) during interference trials with negatively charged emotional words compared to non-interference trials (neutral words) (*p* < 0.05, *Z* ≥ 2.3, Figure 2A, left panel, and Table 1A). Similarly, in the absence of drug ingestion, cStroop significantly activated left middle frontal gyrus (36, 14, 38), right paracingulate gyrus (4, 40, 36), right middle frontal gyrus (48, 20, 42), right frontal orbital cortex (36, 32, 2), left insular cortex (−32, 18, −8), and left inferior frontal gyrus (−48, 14, 6) during interference trials compared to non-interference trials (*p* < 0.05, *Z* ≥ 2.3, Figure 2B, left panel, and Table 1A). We did not find any significant effects of escitalopram in our whole brain analyses.

**Table 1:** **Cluster characteristics**.

	Cluster no.	*Z* max	*X* (mm)	*Y* (mm)	*Z* (mm)	Harvard-Oxford Cortical Atlas	Voxels
**A. WHOLE BRAIN ANALYSES**							
aStroop ctrl	2	4.91	−56	−70	22	52% Lateral occipital cortex, superior division	15714
	1	4.47	−2	−60	10	44% Precuneus cortex	4278
aStroop SSRI	4	4.38	−26	−72	−16	70% Occipital fusiform gyrus	5293
	3	4.12	−54	−16	56	37% Postcentral gyrus	4387
	2	4.3	−42	32	−2	23% Frontal orbital cortex	2667
	1	4.28	0	6	46	32% Juxtapositional lobule cortex	1137
cStroop ctrl	3	4.45	36	14	38	36% Middle frontal gyrus	6140
	2	4.4	−32	18	−2	31% Insular cortex	1973
	1	4.24	42	−46	36	18% Angular gyrus	1943
cStroop SSRI	5	4.43	−54	16	28	42% Inferior frontal gyrus, pars opercularis	10330
	4	4.43	62	−54	−4	79% Middle temporal gyrus, temporooccipital part	7950
	3	4.47	44	20	−12	42% Frontal orbital cortex	6533
	2	4.33	−58	−46	−8	43% Middle temporal gyrus, temporooccipital part	3781
	1	3.99	−14	−74	−46	No label found!	1120
**B. ROI – DLPFC**							
cStroop > aStroop ctrl	1	6.2	46	22	32	40% Middle frontal gyrus	944
cStroop > aStroop SSRI	4	4.67	44	14	20	20% Inferior frontal gyrus, pars opercularis	65
	3	4.17	−40	34	20	29% Middle frontal gyrus	10
	2	4.09	34	10	32	26% Middle frontal gyrus	4
	1	3.94	38	20	26	27% Middle frontal gyrus	2
**C. ROI – ACC**							
cStroop > aStroop ctrl	1	5.01	8	30	38	61% Paracingulate gyrus	66
cStroop > aStroop SSRI	1	3.8	−6	14	46	57% Paracingulate gyrus	4
aStroop > cStroop ctrl	1	3.87	−6	52	2	75% Paracingulate gyrus	3
aStroop > cStroop SSRI	–	–	–	–	–	–	–
(aStroop > cStroop ctrl) > (aStroop > cStroop SSRI)	2	2.95	6	44	12	53% Cingulate gyrus, anterior division	1
	1	2.92	8	44	8	53% Cingulate gyrus, anterior division	1

*The table describes the localizations of the peak voxel in the clusters shown in Figure 2 and the region analyses of dorsolateral prefrontal cortex and anterior cingulate cortex. Cluster forming threshold *Z* ≥ 2.3, corrected *p* = 0.05. The coordinates are in MNI standard. Since the Harvard-Oxford Cortical Atlas is a probabilistic atlas, the anatomical localizations are reported in percentages*.

*aStroop, affective counting Stroop (incongruent > neutral words); cStroop, cognitive counting Stroop (incongruent > neutral words); ctrl, control situation; SSRI, after intake of escitalopram; ROI, region of interest analysis; DLPFC, dorsolateral prefrontal cortex; ACC, anterior cingulate cortex*.

### Region of interest analyses

**Figure 3:**
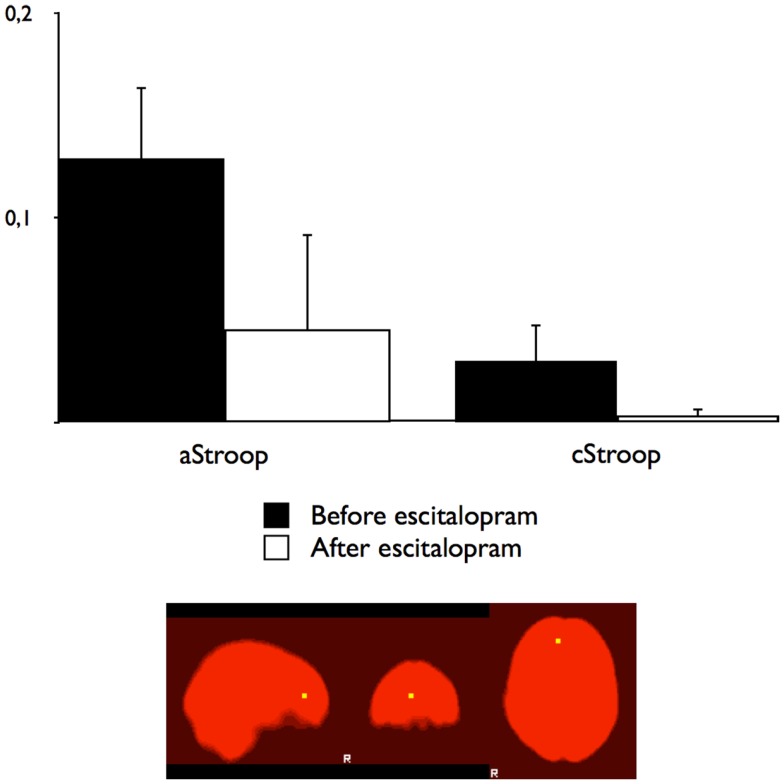
**Blood oxygen level dependant changes in the right ACC**. The BOLD percentage changes in the right rostral anterior cingulate cortex for affective counting Stroop (aStroop) and cognitive counting Stroop (cStroop) before and after intake of escitalopram. The differences were not statistically significant. The mask was smoothed with a Gaussian kernel set to 1.5 mm, surrounding the peak voxel shown in Figure 2A (*x, y, z*: 6, 44, 12).

To compare the effects of escitalopram on aStroop and cStroop in the key nodes of the cognitive control networks, a hypothesis driven ROI analysis was performed *post hoc* in the ACC and the DLPFC. In the absence of escitalopram, aStroop significantly activated rostral ACC compared to cStroop (aStroop > cStroop, corrected *p* < 0.05, Table 1C), while cStroop significantly activated dorsal ACC and DLPFC compared to aStroop (cStroop > aStroop, corrected *p* < 0.05, Tables 1B,C). Ten milligrams of escitalopram decreased the BOLD signal in rostral ACC for the aStroop contrast (aStroop > cStroop, corrected *p* < 0.05, Table 1B), and left intact the BOLD signal in dorsal ACC and DLPFC for the cStroop contrast (cStroop > aStroop, corrected *p* < 0.05, Tables 1B,C). When the effects of escitalopram were compared, the rostral ACC during aStroop turned out to be significantly more activated before compared to after drug intake (corrected *p* < 0.05, Figure 2C). The peak voxel (*x, y, z*: 6, 44, 12) survived a corrected threshold of *p* = 0.05 (at the voxel level, using Gaussian random field theory). The difference was primarily driven by the aStroop after > before escitalopram contrast (Figure 3). No other significant difference was noted in the effects of escitalopram in medial or lateral PFC.

## Discussion

Escitalopram did not change the neural activations of aStroop and cStroop studied separately. However, in support of our hypothesis, an acute dose of SSRI significantly decreased the activation in ACC during aStroop as compared to cStroop. Our finding is consistent with earlier findings, in which increased availability of 5-HT has been shown to reduce the BOLD response in limbic and paralimbic brain regions during exposition to negative emotional stimuli (25).

The voxels that represented the largest difference are located in the rostral part of the ACC. This is in agreement with earlier studies showing that the distribution of 5-HT transporters is denser in the rostral ACC (43) compared to the dorsal ACC, as well as with the putative functional division of the ACC, where rostral ACC is more involved in emotional processing while the dorsal part of the ACC has a more cognitive profile (44).

An interpretation that we favor is that escitalopram by reducing the impact of negative emotions on brain activity made the burden of the affective interference less intense. Our results suggest a mechanism by which SSRI may resolve psychopathological states such as depression or anxiety. Furthermore, our results indicate that SSRI may have an effect already during the initial days of treatment.

It cannot be ruled out that these effects of escitalopram on prefrontal cortex are secondary to effects on other brain regions, such as amygdala, a region in the temporal lobe that is heavily innervated with serotonergic raphe nuclei neurons.

Our study has some limitations. First, the lack of placebo arm is a weakness in our study. We assumed that the placebo effects would be the same in both experimental conditions, aStroop and cStroop, and that a placebo arm therefore would be redundant. However, there may still be some placebo effects remaining such that affects could be more sensitive to placebo effects than cognitive processes. We used the same affective words in all three trials (the training session and the two experimental sessions). To avoid sensitization effects, we used a large number of words (80) and presented the words in a randomized way. We also assumed that if there were any training effects, they would appear during the first few minutes of exposition to the task, i.e., during the training session prior to the experiment. However, it cannot be ruled out that training effects may continue longer than that; our experimental design did not allow us to control for that. Furthermore, we lack behavioral data, due to technical problems with the handheld button box. However, none of the subjects reported any problems in understanding or performing the Stroop tasks, and under control conditions both aStroop and cStroop activated the key nodes of the cognitive control network, the ACC and DLPFC, indicating that a true cognitive control situation induced by the Stroop tasks really occurred. Furthermore, when the first method (cluster corrected whole brain analysis) did not show any significant differences after intake of escitalopram, and we did not want to conveniently change the cluster forming threshold, we chose *post hoc* to do a more anatomically based voxel corrected ROI analysis. Finally, we identify the small number of test persons (*N* = 11) as a limitation to our study.

In future studies, it would be interesting to include patients with known cognitive control impairment and also to investigate whether there are any differences between aStroop and cStroop regarding the sensitivity to training effects and to placebo effects.

## Author Contributions

All authors approved the version to be published, and agreed to be accountable for all aspects of the work in ensuring that questions related to the accuracy or integrity of any part of the work are appropriately investigated and resolved. Christoffer Rahm and Mussie Msghina contributed substantially to the conception and design of the work, the analysis and interpretation of data and the drafting of the work. Benny Liberg contributed to the acquisition, analysis, and interpretation of data and revised the work critically for important intellectual content. Maria Kristoffersen-Wiberg and Peter Aspelin contributed substantially to the acquisition of data and revising the work critically for important intellectual content.

## Conflict of Interest Statement

The authors declare that the research was conducted in the absence of any commercial or financial relationships that could be construed as a potential conflict of interest.
